# Urinary and dietary sodium and potassium associated with blood pressure control in treated hypertensive kidney transplant recipients: an observational study

**DOI:** 10.1186/1471-2369-13-121

**Published:** 2012-09-26

**Authors:** Annie Saint-Remy, Mélanie Somja, Karen Gellner, Laurent Weekers, Catherine Bonvoisin, Jean-Marie Krzesinski

**Affiliations:** 1 Nephrology-Hypertension Unit, University Hospital of Liege, Liege, Belgium; 2 Dietetics Unit, University Hospital of Liege, Liege, Belgium; 3 Krankenhaus Düren-Medizinische Klinik II, Düren, Germany

**Keywords:** Kidney transplantation, Office blood pressure, Home blood pressure, Sodium, Potassium, Urinary sodium/potassium ratio

## Abstract

**Background:**

In kidney transplant (Kt) recipients , hypertension is a major risk for cardiovascular complications but also for graft failure. Blood pressure (BP) control is therefore mandatory. Office BP (OBP) remains frequently used for clinical decisions, however home BP (HBP) have brought a significant improvement in the BP control. Sodium is a modifiable risk factor, many studies accounted for a decrease of BP with a sodium restricted diet. Increased potassium intake has been also recommended in hypertension management. Using an agreement between office and home BP, the present study investigated the relations between the BP control in Kt recipients and their urinary excretion and dietary consumption of sodium and potassium.

**Methods:**

The BP control defined by OBP <140/90 mmHg and HBP <135/85 mmHg was tested in 70 Kt recipients (mean age 56 ± 11.5 years; mean graft survival 7 ± 6.6 years) treated with antihypertensive medications. OBP and HBP were measured with a validated oscillometric device (Omron M6®). The 24-hour urinary sodium (Na+) and potassium (K+) excretions as well as dietary intakes were compared between controlled and uncontrolled (in office and at home) recipients. Non parametric Wilcoxon Mann–Whitney Test was used for between groups comparisons and Fisher's exact test for frequencies comparisons. Pearson correlation coefficients and paired t-test were used when sample size was >30.

**Results:**

Using an agreement between OBP and HBP, we identified controlled (21%) and uncontrolled recipients (49%). Major confounding effects susceptible to interfere with the BP regulation did not differ between groups, the amounts of sodium excretion were similar (154 ± 93 vs 162 ± 88 mmol/24 h) but uncontrolled patients excreted less potassium (68 ± 14 vs 54 ± 20 mmol/24 h; P = 0.029) and had significantly lower potassium intakes (3279 ± 753 vs 2208 ± 720 mg/24 h; P = 0.009), associated with a higher urinary Na+/K + ratio. Systolic HBP was inversely and significantly correlated to urinary potassium (r = −0.48; P = 0.002), a positive but non significant relation was observed with urinary sodium (r = 0,30;P = 0.074).

**Conclusions:**

Half of the treated hypertensive Kt recipients remained uncontrolled in office and at home. Restoring a well-balanced sodium/potassium ratio intakes could be a non pharmacological opportunity to improve blood pressure control.

## Background

Hypertension (HT) is highly prevalent among kidney transplant recipients (Kt), as 60 to 80% or more are concerned and the proportion of hypertensive patients receiving medication increases with time after kidney transplantation [1,2]. Hypertension is not only a major risk for cardiovascular (cv) complications but also for graft failure since a systolic blood pressure of 140 mmHg or higher was associated with a significant graduated increase of graft failure [3]. This suggests a great importance to control blood pressure (BP). Although Office BP (OBP) remains certainly the most frequently method used for clinical decisions, complementary measurement techniques such as 24 h ambulatory blood pressure monitoring (ABPM) and home blood pressure (HBP) have brought a significant improvement in the identification of BP abnormalities and BP control in Kt recipients also [4,5]. These techniques appear to be more reproducible and superior in predicting target organ damage and cardiovascular events than OBP [6,7]

Many factors may be involved in the onset of HT in Kt, among them the classical ones found in the general population such age, Body Mass Index (BMI), diabetes, kidney function, and those specifics to the transplantation, the donor's characteristics, the graft quality, immunosuppressive therapy with calcineurin inhibitors and corticosteroids.

Salt (sodium chloride) is a modifiable risk factor and although controversial, its relation with blood pressure and cardiovascular disease has been largely documented [8,9]. Excessive salt consumption concerns many countries, so World Health Organization has promoted a less than 6 g daily intake to prevent stroke and heart disease [10]. Increased potassium intake has also been recommended for prevention and treatment of hypertension, especially in those who are unable to reduce their intake of sodium [11,12].

As many Kt recipients with hypertension require several medications to reach target BP, it is of importance to add lifestyle modifications as a support contributing to the decrease of BP.

In the present study, we analyzed the quality of BP control and the relations between this control defined by office and home BP in Kt recipients treated with antihypertensive medications and their 24-h urinary sodium (Na+) and potassium (K+) excretion as well as their corresponding dietary intakes obtained by dietary recall.

## Methods

### Study sample

Two hundred and eleven Kt received a mail with the objectives of the study. Taking into account the early period after transplantation during which one many physiological and medical treatment modifications can arise, Kt recipients with a transplantation less than 1 year were excluded. On their next scheduled visit at the hospital, patients had the opportunity to receive more complete details on the study if necessary, those who agreed were invited to sign the informed consent. So a total of 78 patients with stable allograft function were included. As the study focuses on BP control under medications, 8 of these patients who were not treated with antihypertensive drugs were excluded.

The study received the approval from the Comité d'Ethique Hospitalo-Facultaire Universitaire de Liège.

### Blood pressure measurements

BP measurements were first done at the hospital (office BP). At the baseline visit, patients had to bring a 24 h urine collection and a completed report about what they ate and drunk during the urine collection period. After recording the clinical current conditions and the medications OBP was measured with an oscillometric automatic device OMROM M6® and adapted cuff size if necessary. OBP represents the mean of 3 consecutive measurements. If the 2 last measurements still varied from more than 5 mmHg, additional measurements were done.

### Home blood pressure

After the baseline visit, all patients received explanations on how to perform correctly home BP measurements (HBP), with an OMROM M6® that they have to bring back at the end of one week, usual recording period.

They had to record their HBP during 7 days with 2 measurements in the morning and 2 in the evening. HBP were averaged taking in account measurements from the day 2 to the day 7 [13].

### Hypertension criteria

Hypertension was defined when office BP was ≥ 140 and/or ≥ 90 mmHg (≥ 130 and/or ≥ 80 if diabetes) and when HBP was ≥ 135 and/or ≥ 85 mmHg (≥ 130 and/or ≥ 80 if diabetes) [13].

The antihypertensive treatment was not allowed to be changed during the study.

### Urine collection

Two 24 h urine collections were available, the first one at the baseline visit and a second one when patients came back to the hospital at the end of the one week HBP recording period. Although accustomed to urine collection, patients were told again how to proceed.

Diuresis, sodium/24 h (mmol/24 h), potassium/24 h (mmol/24 h) and creatinine/24 h (g/24 h) were measured and sodium to potassium ratio (Na/K) was calculated. Proteinuria was also measured on this urine collected.

### Dietary intake

They also brought, as at the start of the study, with the second time 24 h urine collection, a detailed description of food and beverages consumed during urine collection, allowing the calculation of sodium and potassium intake during both 24 h periods. Quantitative analysis of the 24 h food records was done with the Belgian food table: NUBEL [14]. When a diet product was not referred in the NUBEL table, composition was extracted from the product itself if present or was analyzed with the composition table from the Paul Lambin Institute [15], or according to Souci et al. [16]. A particular attention was held to the bottled water consumed by patients because some of them were rich in sodium and to the water distribution whose composition could differ according to the living place of the patients. For a simple estimation of added salt, patients were asked if they used salt when cooking and/or when they ate.

### Statistical analysis

Continuous data are presented as means (± standard deviation) and frequencies as percentage.

According to the groups sample size, non parametric Wilcoxon Mann–Whitney Test was used for between groups comparisons (Tables 1 and 2) and the Fisher's exact test (two tailed) was used for percentage comparisons (Tables 1, 2 and 3). Pearson correlation coefficients, partial correlation coefficients (Table 4) and paired t-test were used when sample size was >30 (Table 5). Two-sided P <0.05 was considered as statistically significant.

**Table 1:** Characteristics of patients according to the control of Office Blood Pressure

	**OBP <140 and <90 (mmHg)**	**OBP ≥140 and/or ≥90 (mmHg)**	**P**
N	29	41	
Age (years)	56 ± 11	57 ± 11.7	0.70
Graft survival (years)	7.5 ± 7.3	6.4 ± 6	0.49
Office SBP (mmHg)	126 ± 9	145 ± 11	<0.00001
Office DBP (mmHg)	78 ± 8.5	86 ± 13	0.004
Heart rate (beat/min)	68 ± 10	69 ± 12	0.80
Hemodialysis vintage (years)	3.0 ± 5.4	2.5 ± 1.7	0.56
BMI (kg/m^2^)	25.7 ± 4.7	25.8 ± 4.7	0.92
Number of antihypertensive drugs	2.1 ± 1	2.1 ± 1	0.94
GFR(ml/min)	68.2 ± 25	63.1 ± 25	0.23
Calcineurin inhibitors N (%)	26 (89.6)	38 (92.7)	0.68
Prednisone (daily dose)	4.7 ± 1.8	4.7 ± 1.0	0.87
Urinary excretion			
Diuresis (ml)	2439 ± 846	2593 ± 968	0.50
Na^+^ (mmol/24 h)	155 ± 72	176 ± 90	0.33
K^+^ (mmol/24 h)	66 ± 23	59 ± 25	0.28
Na^+^/K^+^	2.5 ± 1.1	3.2 ± 1.2	0.035
NaCl (g/24 h)	9.1 ± 4.2	10.3 ± 5.3	0.32
Diet intakes			
Na^+^ (mg/24 h)	2289 ± 942	1868 ± 679	0.17
K^+^ (mg/24 h)	2900 ± 780	2243 ± 773	0.025

**Table 2:** Comparisons between controlled and uncontrolled patients- reference to office and home BP

	**OBP < 140 and <90 and HBP < 135 and <85**	**OBP ≥140 and/or ≥90 and HBP≥135 and/or ≥85**	**P**
N	15	34	
Age (years)	53.4 ± 9.8	56.8 ± 11	0.33
Graft survival (years)	5.3 ± 4	6.3 ± 6	0.82
Nephrectomy (native kidney), (N)	1	5	0.65
Donor			
Age (years)	39.9 ±12.4 [17–54]	42.7 ±16.5 [15–67]	0.44
Hypertension, (N)	3	5	0.69
Cadaveric, (N)	14	34	0.31
Living, (N)	1	0	0.31
Office SBP (mmHg)	125 ± 9	146 ± 12	0.000002
Office DBP (mmHg)	77 ± 8	87 ± 13	0.004
Office heart rate (beat/min)	67 ± 9	69 ± 12	0.78
Home SBP (mmHg)	123 ± 7	141 ± 10	0.000001
Home DBP (mmHg)	77 ± 6	85 ± 11	0.0005
Home heart rate (beat/min)	68 ± 5	70 ± 12	0.81
Hemodialysis vintage (years)	4.0 ± 7	2.6 ± 2	0.44
BMI (kg/m^2^)	24 ± 4.6	26 ± 4.9	0.18
Number of antihypertensive drugs	2.1 ± 1	2.1 ± 1	0.99
GFR(ml/min)	63.2 ± 28	61.5 ± 21	0.94
Calcineurin inhibitors N(%)	14 (93)	31 (91)	1.00
Cyclosporine	2 (13)	14 (41)	0.045
Tacrolimus	12 (80)	17 (50)	
Prednisone (daily dose)	3.9 ± 1.2	4.7 ± 1	0.09
K + (blood;mmol/l)	3.9 ±0.3 [3.1-4.5]	4 ± 0.4 [3.2-5]	0.56
Urinary excretion			
Diuresis (ml)	2431 ± 719	2457 ± 919	0.90
Na^+^ (mmol/24 h)	154 ± 93	162 ± 88	0.60
K^+^ (mmol/24 h)	68 ± 14	54 ± 20	0.029
Na^+^/K^+^ ratio [CI 95%]	2.3 ± 1.2 [1.6-3.1]	3.2 ± 1.3 [2.7-3.6]	0.052
NaCl (g/24 h)	9.1 ± 5.4	9.6 ± 5.2	0.60
Diet intakes			
Na^+^ (mg/24 h)	2339 ± 1067	1766 ± 695	0.23
K^+^ (mg/24 h)	3279 ± 753	2208 ± 720	0.009

**Table 3:** Etiology of renal disease, classes of antihypertensive medications and proteinuria

	**OBP < 140 and <90 and HBP < 135 and <85**	**OBP ≥140 and/or ≥90 and HBP≥135 and/or ≥85**	**P**
N	15	34	
Etiology of renal disease. N (%)			
Vascular disease	0	1 (3)	1.00
Glomerulonephritis	4 (26.7)	11 (33.3)	0.75
Polycystic kidney disease	4 (26.7)	6 (18.2)	0.47
Diabetic nephropathy	1 (6.7)	4 (12.1)	1.00
Tubulo intertitial disease	1 (6.7)	6 (18.2)	0.22
Unknown	5 (33.3)	5 (15.2)	0.13
Antihypertensive drugs N (%)			
Diuretics	4 (26.7)	10 (29)	1.00
Thiazide and loop	3	9	0.73
K-sparing	1	1	0.51
Beta-blockers	9 (60)	21 (61.8)	1.00
Calcium-channel blockers	9 (60)	18 (53)	0.76
Angiotensin converting enzyme inhibitors	5 (33.3)	11 (32.3)	1.00
Angiotensin receptor blockers	4 (26.7)	5 (14.7)	0.43
Centrally acting sympathicolytics	1 (6.7)	8 (23.5)	0.24
Proteinuria (mg/g creatinine)			
<40	3 (21.4)	9 (26.5)	0.73
40–200	10 (71.4)	19 (55.9)	0.35
>200	2 (13.3)	6 (17.6)	1.00

**Table 4:** Coefficients of partial correlation between urinary Na + and K + excretion and Home systolic blood pressure

**Pearson correlations**	**Partial correlation coefficient**	**P**
N = 49		
Home SBP with urinary Na+ Controlled for: age, BMI, smoking habit, antirejection drugs and urinary K+	0.30	0.074
Home SBP with urinary K+ Controlled for: age, BMI, smoking habit, antirejection drugs and urinary Na+	- 0.48	0.002

**Table 5:** Comparisons of electrolytes excretion and diet intakes 7 days apart

	**24 h urine collection at baseline visit mean ± sd**	**24 h urine collection 7 days later mean ± sd**	**P**
N = 49			
Diuresis	2455 ± 894	2495 ± 925	0.60
Urinary Na + (mmol/24 h)	160 ± 89	157 ± 72	0.23
Urinary K + (mmol/24 h)	58.2 ± 19	58.7 ± 22	0.69
Diet Na + (mg/24 h)	2008 ± 892	2217 ± 882	0.86
Diet K + (mg/24 h)	2659 ± 896	2814 ± 873	0.50

Statistical analyses were performed with the Statistica® v10 software ( http://www.Statsoft.com).

## Results

Baseline characteristics of the treated hypertensive Kt are described in Table 6.

**Table 6:** Baseline characteristics of patients

	**Mean ± sd [min-max]**
N	70
Men/women (N)	43/27
Age (years)	56 ± 11.5 [33–76]
Graft survival (years)	7.0 ± 6.6 [1-25]
Hemodialysis vintage (years)	2.7 ± 3.7 [1 month-7.9 y]
GFR (ml/min)	65.6 ± 24 [26–133]
BMI (kg/m^2^)	25.8 ± 4.7 [16-37]
BMI ≥30	15 (21%)
Diabetes (N,%)	19 (27)
Current smokers (N,%)	9 (13)
Office SBP (mmHg)	136 ± 14 [107–175]
Office DBP (mmHg)	83 ± 12 [50–108]
24 h urinary Na + (mmol)	167 ± 83 [45–463]
24 h urinary K + (mmol)	62 ± 24 [25–134]
NaCl (g/24 h)	9.9 ± 4.9 [2.6-27]
Urinary Na+/K + ratio	2.9 ± 1.2 [0.8-6]

Mean age was 56 (± 11.5) and patients were transplanted for 7 years (± 6.6), the mean GFR (Cockcroft-Gault formula) was 65.6 (± 24) ml/min. Before kidney transplantation, they were all hemodialyzed with a mean time of 2.7 years. Obesity (BMI ≥30 kg/m^2^) was present in 21% and diabetes frequency was 27%, the majority (90%) were transplantation induced diabetes. Patients were treated by a mean number of 2 antihypertensive drugs (± 1). The 24 h mean urinary NaCl excretion was around 10 g indicating a high salt consumption equal to the one observed in the Belgian population and far from the 5 g recommended by the World Health Organization [17].

### Sodium, potassium and control of office blood pressure

The 70 patients with an antihypertensive treatment have been divided into 2 groups according the office BP control. Uncontrolled patients were defined by an OBP ≥ 140 and/or 90 mmHg (≥ 130 and/or ≥ 80 if diabetes), so 41 patients (59%) remained hypertensive despite the treatment while 29 (41%) had their BP well controlled. Among uncontrolled patients, 56% were men.

Comparisons between the 2 groups of controlled and uncontrolled office BP did not identify any statistical difference for age, graft survival, HD vintage, BMI, GFR, proportion of treated patients with calcineurin inhibitors or the mean number of antihypertensive drugs taken daily (Table 1). Logically, mean office systolic and diastolic BP of uncontrolled patients were significantly higher. Both groups had a similar salt consumption nearby 10 g/day. However, the urinary Na/K ratio was significantly lower for patients with controlled BP than those uncontrolled (2.5 vs 3.2). Analysis of diet records shew that both groups did not differ in their salt consumption but controlled patients consumed higher amounts of potassium by regularly eating more fruits and vegetables, this could be in accordance with their lower observed Na+/K + ratio.

### Sodium, potassium and control of office and home blood pressure

Did home BP bring any additional value to the association observed between office BP control and urinary excretion of sodium and potassium? To answer that question, the initial sample of 70 treated patients was classified not on the only OBP control but on the OBP and HBP control. So, uncontrolled patients were defined when their OBP was ≥ 140 and/or ≥90 mmHg (≥ 130 and/or ≥ 80 if diabetes) and their HBP was ≥ 135 and/or ≥85 mmHg (≥ 130 and/or ≥ 80 if diabetes), in other words when despite the treatment they remained hypertensive in the office and at home. Patients were defined as well controlled when OBP and HBP were lower than the above mentioned threshold criteria.

Controlled patients were 15 (21%) and uncontrolled in both conditions were 34 (49%) (Table 2). Masked hypertension (normal OBP but hypertension at home) was identified in 15 (21%) and white coat hypertension (high OBP but normal BP at home) concerned 6 patients. As when considering the office BP only, the comparisons between controlled and uncontrolled patients both in office and at home did not underline significant differences for major parameters except for OBP and HBP of course. Donor's risk factors such as age or presence of hypertension did not differ. In both groups the majority were transplanted with a cadaveric kidney. Only 6 patients (one controlled and 5 uncontrolled) had a monolateral nephrectomy, so, among the 49 patients with true controlled or uncontrolled hypertension, 43 had their two native kidneys yet.

Classifying patients with that other method based on an agreement between office and home BP, generated also a group of uncontrolled patients whose urinary Na + excretion did not differ from the one of controlled but they significantly excreted less K + (P = 0.029). Their higher urinary Na+/K + ratio was just at the limit of statistical significance, however it seemed that they clearly consumed less potassium than controlled patients (P = 0.009), measured by the dietary recall technic. The same results were observed when the sodium/creatinine ratio was used.

There were no differences according the causes of renal insufficiency (Table 3) and the two groups were treated with similar proportions of the different classes of antihypertensive medications. Finally, proteinuria did not show any difference between groups.

It is important to note that the proportion of patients treated with diuretics (majorly thiazide and loop diuretics in both groups) did not differ between groups, 26.7% of controlled and 29.4% of uncontrolled. To verify any impact of the use of such diuretics on our observations mainly on the electrolytes excretion, we performed an internal comparison of OBP, HBP, urinary Na + and K + inside the two groups, controlled and uncontrolled, between those treated with or without a diuretic. No statistical difference for these parameters were observed between patients treated with or without diuretic drugs either in controlled or in uncontrolled patients. Moreover, let us remind that no change in the diuretic doses was allowed 1 month before the study and during the week of HBP measurement.

The observed association between uncontrolled BP and a lower potassium excretion has been tested by Pearson correlation on the 49 patients (true controlled and uncontrolled) among the 70 (Table 4).

Partial correlation coefficients have been calculated with systolic HBP by controlling age, BMI, antirejection drugs and smoking habit. Concerning the relation between urinary Na + excretion and systolic HBP, the correlation has been also controlled for the urinary K+. A positive correlation was observed (0.30, P = 0.074) however the coefficient did not reach statistical significance. On the other hand, when controlled for urinary Na + excretion, a negative and highly significant relation was noted indicating an inverse association between the systolic HBP and excretion or intakes of potassium. However, no such results were observed with diastolic BP.

### Reproducibility of urinary sodium and potassium

As two 24 h urine collections were available (at baseline visit and at the end of HBP recording) as well as food intakes during urine collection, a comparison of urinary and diet Na + and K + has been performed on the sample to assess the short term reproducibility of these parameters (Table 5).

Knowing that no change in the antihypertensive treatment occurred during the study, the paired comparisons between first and second urine collection did not show any statistical difference concerning urinary Na + and K + excretion as well as Na + and K + intakes which remained very similar into that period of time.

## Discussion

### Blood pressure

Controlling BP in hypertensive kidney transplant patients is mandatory to prevent cardiovascular complications and graft failure [18]. In most patients, several drugs are needed to reach a BP lower than 140/90 mmHg (or 130/80). In such particular patients with a single functioning kidney, BP control can be difficult to reach because of a restricting therapy including immunosuppressive and corticosteroid medications. Our purpose was to analyze the daily practice control of BP by two methods (OBP and HBP) and the factors which could influence such control, especially looking for the potential contribution of non pharmacological intervention such to restore a well-balanced sodium/potassium ratio intakes in treated by antihypertenvive drugs Kt patients, as it was described in non transplanted hypertensive population [19,20].

Indeed, in Kt patients with a stable kidney function, taking into account several determinants of BP, even though controlled and uncontrolled patients seemed to consume both a similar amount of salt, the controlled patients had significantly higher intakes of potassium, this could be associated with their lower Na/K ratio compared to uncontrolled patients. On one hand, when controlled for age, BMI, antirejection drugs, smoking habit and Na + excretion, an inverse and significant relation was found between urinary potassium and HBP. On the other hand a positive but non significant relationship was observed between urinary sodium and HBP when controlled for K + excretion. As for many patients, out-of-clinic BP are recommended for Kt patients [21,22]. In the present study, we favoured the use of two complementary BP measurement techniques i.e. office and home and defined controlled or uncontrolled BP when there was an agreement between both techniques. That classification of BP reinforced their truly status of normotensive or hypertensive treated patients taking into account the well known limitations of office BP and allowed the detection of masked and white coat hypertension [23].

The frequency of Kt patients remaining hypertensive despite a treatment requiring on average 2 or more drugs is too high, 58.6% based on OBP only but it decreases to 49% when based on both OBP and HBP (after excluding in the calculation of the proportion other forms such white-coat and masked hypertension).

Interpreting BP level in Kt patients is complex since many factors are susceptible to modify BP regulation.

Age, and BP status of donor, presence of native kidneys in recipients or number and kind of antihypertensive drugs did not differ between uncontrolled and controlled patients.

### Imunosuppressive medications

Our controlled patients are mainly treated with tacrolimus (80%) (Table 2) compared with uncontrolled ones (50%) and this could partly explain the higher BP of the uncontrolled group as cyclosporine has been considered as a more hypertensive drug [24]. However in our study, on average, the number of antihypertensive drugs did not differ between the two BP control groups, moreover a comparison of BP (office and home) and of the number of antihypertensive drugs between patients treated by either cyclosporine or tacrolimus did not show any statistical difference. On average, our patients were transplanted for 5 (controlled) and 6 years (uncontrolled), it is known that incidence of hypertension increases with increasing time after transplantation, this is partly due to additional risk factors but also to the deleterious effects on the cardiovascular system caused by the sum of pre and posttransplant risk factors, so at this stage, it is difficult and even hazardous to attribute the uncontrolled BP to the only higher proportion of patients treated with cyclosporine rather than tacrolimus. Even if patients treated with tacrolimus were transplanted more recently than those treated with cyclosporin (P = 0.03), there were no significant differences according to age, GFR and time duration spent on hemodialysis.

Steroid treatment has been said to contribute to 15% in hypertension [25]. The frequencies of patients still treated with steroid were similar in cyclosporine and tacrolimus treated patients. Adherence to medications is also a key point in reaching BP target, all the patients questioned on their regularity in taking medications were particularly convinced by the importance of immunosuppressive and antihypertensive treatments regarding the graft protection.

### Urinary and dietary sodium and potassium

Urinary Na + excretion is considered as representative of the dietary intakes in stable conditions. On average, in our study, controlled patients did not excrete less Na + than uncontrolled ones. The mean salt consumption was however between 9 and 10 g per day, with 87% who had salt consumption higher than 5 g, so as in many countries, mean values exceeded largely the physiological needs [26] and this in spite of the fact that there are hypertensive treated by antihypertensive drugs.

Calculated sodium intakes were lower than urinary excretion. The difference can be explained by the lesser precision of the 24-h dietary recall intakes declared by patients recording their diet intake while they were collecting urine. Indeed, the real intakes were most certainly underestimated because the exact amount of food eaten by the patients was very difficult to know and moreover we could not assess the salt added in cooking and at table. However the patients were asked if they systematically added salt when cooking and/or at table, so 8 (53%) controlled patients admitted to add salt against 22 (65%) uncontrolled patients. Next the added salt, bread, high salted cheese and cooked meats and processed food were major diet sources of salt. Interestingly, the intakes and electrolytes excretions were reproducible on two occasions, 7 days apart. As sodium intake was less accurately quantified by dietary recall, a 24 h urine collection measuring sodium excretion would be better, after explaining the patient how to proceed.

On the contrary, the agreement between diet and urinary potassium seemed clearly better. This was documented in the study of Tasevska et al. who found a high correlation (r = 0.89) between dietary potassium and its urinary excretion in free living individuals [27].

Although all patients had potassium intakes lower that the 4700 mg recommended [28], patients with controlled BP had on average higher potassium intakes with a diet richer in fruits and vegetables and contrary to what is generally said i.e. the high sodium consumers consume less potassium, in our population, there was no inverse relation between sodium and potassium intakes. The low consumption of potassium for several patients was not necessarily an individual's preference for salted food but was probably an habit they have kept when they were treated by hemodialysis, a situation in which strict limitations of potassium intakes are mandatory.

Randomized large-scale control trials on the effect of a modified sodium and potassium diet on blood pressure in late kidney transplant patients are lacking, however dietary sodium restriction (80–100 mmol/day or 1.8 to 2.3 g/day) is recommended to contribute to the cardiovascular burden management and also to limit the cyclosporin-induced hypertension caused by sodium retention in early post-transplantation [29].

Although the legendary salt controversy is still a hot topic [30-34], numerous studies favored a beneficial effect on BP, even sometimes modest, with reduction of salt intake and increasing potassium intakes. A drop of 3.5/2.5 mmHG with a 1.7 g increase of potassium intake and a drop of 5.2/3.7 mmHg with a decrease of 4.5 g of NaCl could be reached, however effects were larger in hypertensive than normotensive individuals [35]. Potassium supplementation was associated with a small but significant reduction in mean systolic and diastolic blood pressure ,-3.11 mm Hg and −1.97 mm Hg respectively. Effects of treatment appeared to be enhanced in studies in which participants were concurrently exposed to a high intake of sodium [11]. The beneficial effect of K on BP could be explained by a vasodilation action due to hyperpolarization of endothelial and vascular smooth muscle cell of the arterial wall [36]. Moreover, high potassium intake can decrease the salt sensitivity and also decrease the need for antihypertensive drugs [37].

Some observational studies did not find a correlation between salt intakes, estimated by 24-h urinary Na+, and prevalence of hypertension or BP in Kt recipients with stable allograft function [38,39]. In these studies, measured office BP or hypertension defined by the prescription of antihypertensive medication, and the number of drugs were considered as a surrogate marker for severity of hypertension and no dietary analysis of sodium and potassium intakes was performed. In agreement with Prasad [40], we did not find any statistical difference of BP between patients treated with cyclosporine or tacrolimus and no difference in patients treated with or without a thiazide diuretic. However some methodological differences could explain our conclusions, indeed we defined the BP control on the basis of two measurement techniques and 24-h Na + and K + measurements were associated to dietary intakes assessments. We observed a positive but non significant relation between urinary sodium and systolic home BP when confounding factors such as age, BMI, antirejection drugs, smoking habit and potassium excretion were controlled. We also observed a significantly negative correlation between systolic home BP and potassium excretion when age, BMI, antirejection drugs, smoking habit and sodium excretion were controlled. Therefore our results plead in favor of a possible efficient non pharmacological intervention by restricting sodium intakes and increasing potassium ones in Kt recipients. This was documented for sodium in the study of Keven et al. who measured a decrease in BP with daily intakes limited to the recommended levels between 80 and 100 mmol [41]. In a very recent paper [42], van den Berg et al. found that sodium intake was positively and significantly associated with systolic and diastolic office BP in renal transplant recipients compared to healthy controls. Authors concluded that decreasing sodium intake to recommended amounts could decrease systolic BP by 4–5 mmHg. Conversely it was noted in CKD patients that high salt intake can increase proteinuria and ESRD risk [43]. In our study, proteinuria was not correlated with urinary Na excretion, but we have not tested the specific effect of a modification of such sodium intake on the proteinuria. This should be done to reinforce the argument for lowering sodium intake especially in hypertensive Kt patients who are proteinuric and exposed to higher cardiovascular and kidney disease progression risks.

Our uncontrolled patients had a higher Na+/K + ratio than controlled ones. The risk of subsequent cardiovascular disease related to a high Na+/K + ratio has been identified in adults with prehypertension, the effect of the ratio was even stronger than that of sodium or potassium alone [44]. It has to be confirmed in our Kt population with a prospective study.

Our study limitations concern the small size of the sample notably due to our selection criteria to define controlled and uncontrolled patients based on two measurement techniques in late Kt recipients. Cases of masked and white coat hypertension are not discussed in the present paper and have to be studied apart since interestingly, in the context of our study, we did not find a significant relation between sodium, potassium excretion and BP in those particular forms of hypertension (data not shown). Either confounding factors or other determinants of BP level in these patients have to be identified. Our study shows that measurements of Na + and K + intakes with a diet recall fulfilled by patients in a free way leads to limitations when data are compared with urinary excretion. We think that the main limitations came from patients who did not mentioned their complete intakes while they were collecting 24 h urines. Another problem came from the estimation of real amounts of food intakes which were not always accurately described by several patients, so we had to use standardized portions defined by “Poids et mesures- quantification standardisée des denrées alimentaires (Conseil Supérieur d'Hygiène, janvier 2005). The discrepancy between intakes and urinary Na + is higher than with K, it can be explained by the fact that added salt was impossible to measure and that the amount of sodium in some processed foods was not mentionned, this was coupled with possible omissions from the patients in their recall diet document. The better correlation between intakes and urinary excretion of K + has been described in literature. Cooking methods are more conservative for potassium and added potassium sources are much more limited than those for sodium. It is possible that patients have been more reliable when they noticed their food consumption containing potassium rather than the one containing sodium. Obviously, those patients have kept habits from the time they were treated by dialysis when potassium intakes were strictly limited that is probably why they do not consume fruits and vegetables enough. We recognize that these considerations bear witness of a limited use of diet recall to calculate amounts of sodium and potassium. This has been mentionned in literature, so patients tend to underestimate their Na + intake by 30 to 50% while estimated K + intake better correlated with 24-h urinary excretion [45]. These observations are in favour of the use of 24 h urinary excretion provided that renal function and medications have to be taking in account.

Our study is crossectional so it gives a snapshot of a situation influenced by the complex and atypical risk factors profile of Kt patients mixing traditional, predisposing and associated to transplantation risk factors. Moreover, the factors which contribute to elevated BP vary between the early and late posttransplant periods, conditioning a continuous increase of the cardiovascular risk throughout time. Our patients have a mean transplantation duration of 7 years (± 6.6), so in that late posttransplant period, next to the allograft function supervision, the management of the cardiovascular risk factors, among them hypertension, can beneficiate of the acquired experience with notably non pharmacological interventions in general population.

## Conclusions

Our observational study shows that a large proportion of Kt recipients treated by drugs for hypertension present an uncontrolled BP using two different technics for BP measurements. Moreover, although both controlled and uncontrolled patients excreted a similar quantity of sodium in their urine, the uncontrolled patients excreted less potassium and had a higher Na+/K + ratio than controlled patients. The home systolic BP, but not diastolic, was inversely and significantly correlated to urinary potassium when age, BMI, antirejection drugs, smoking habit and urinary sodium were controlled.

However, at this stage according to limited accuracy of diet recall, we cannot prove that in late kidney transplant recipients treated with antihypertensive drugs, there is a link of causality between the control of BP and the sodium/potassium balance intakes. To do this, a randomized control trial on a larger sample of patients is needed. However, our results support the idea of a potential relation between BP and the urinary and possibly the dietary sodium/potassium ratio in treated hypertensive uncontrolled patients with only mild CKD, suggesting so a non pharmacological opportunity to improve the BP control. This requires a validation of the diet recall technic and a randomized study to test this hypothesis especially in those who are in the mild form of kidney disease.

## Competing interests

None of the authors has competing interests to declare.

## Authors' contributions

ASR: conception, design, acquisition of data, analysis and interpretation. MS : analysis of diet data and interpretation. KG : acquisition of data, has given final approval of the version to be published. LW : substantial contribution to acquisition of data and their interpretation. CB : substantial contribution to acquisition of data and their interpretation. JMK: has revised the manuscript critically for important intellectual content. All authors read and approved the final manuscript.

## Pre-publication history

The pre-publication history for this paper can be accessed here:

http://www.biomedcentral.com/1471-2369/13/121/prepub

## Aknowledgements

We are grateful to Mrs Marie-Antoinette Graceffa and Mrs Michelle Focan, study nurses and Mrs Jacqueline Barbier, secretary, from the Nephrology-Hypertension Unit, for their useful contribution to the organization of the present study.
